# Share If You Believe, Comment If You Doubt: The Effect of Source of Information, Trust, and Belief in Conspiracy Theories on Engagement with Facebook Posts

**DOI:** 10.3390/bs14080673

**Published:** 2024-08-03

**Authors:** Erga Atad, Yossi David

**Affiliations:** 1 Lauder School of Government, Diplomacy and Strategy, Reichman University, Herzliya 4610101, Israel; 2 School of Communication, Bar-Ilan University, Ramat Gan 5290002, Israel; 3 Department of Communication Studies, Ben-Gurion University of the Negev, Beersheba 8410501, Israel; davidyos@bgu.ac.il

**Keywords:** sharing, communication behavior, crisis communication, trust, belief in conspiracy theories

## Abstract

This study examines the effect of one of three sources of information: a politician (authority figure), a physician (expert), and an ordinary person (non-expert) who appeared in a personal story related to a controversial issue (COVID-19 vaccination) on Facebook, on the willingness to engage with it. Using a between-subjects experiment (N = 848) conducted among Israeli adults (18 and older), we found a higher likelihood of sharing the story in interpersonal conversations than in other types of communications, regardless of the source that appeared in the story. However, respondents with high levels of institutional trust preferred sharing a politician’s story, while conspiracy believers tended to comment on an ordinary person’s story. The findings of the different patterns of communication behavior among conspiracy believers and people with high trust in political institutes contribute to our understanding of the mechanisms underlying the spread of misinformation in the digital age and during times of crisis.

## 1. Introduction

Social media platforms, particularly Facebook, are central domains that provide information about everyday life and emergencies. For example, during the COVID-19 pandemic, many Israelis (60%) used social media to stay informed [1,2]. At the same time, the vast majority (63%) reported difficulties distinguishing trusted news from fake news and reported higher levels of skepticism about the accuracy of the news that politicians shared on social media platforms [1,2].

Using digital platforms like Facebook, users can interact with information via features such as likes, comments, and shares [3]. However, there is a variety in using such actions, and many prefer liking content rather than sharing it [4]. During times of crisis, social media engagement can provide insight into public sentiment towards and the level of trust in government authorities. Times of crisis, such as the health crisis, have generated a high volume of social media discussion globally [2,5,6]. However, at the same time, public opposition to official guidelines and health guidelines led to the spread of misinformation and conspiracy theories [7], which resulted in vaccine hesitancy in various countries [8,9].

Previous studies associated social media usage during crises with lower trust in content [10,11,12], the spread of conspiracy theories [13,14], and decreased trust in governmental entities, which affected vaccine hesitancy and refusal [15,16]. However, this paper aims to examine a neglected topic in these studies: manipulating the identity of the person who appeared in a personal story on a Facebook post to understand how such identity affects the willingness to engage with the story. We aim to explore how exposure to social media stories impacts communication behavior concerning the users’ institutional trust and belief in conspiracy theories. Based on [17] (p. 24), we defined conspiracy theories as ‘manipulating and misleading people intentionally to achieve political ends’. To examine this effect, we conducted an experimental study (N = 848) in December 2021 to explore user engagement (likes, comments, and shares) with personal stories on Facebook posts using a four-condition, including a personal story published by (1) a politician, (2) a physician, and (3) a citizen, as well as (4) a neutral story.

This study suggests that such an examination is necessary to understand users’ communication behaviors and their potential consequences on health behaviors during a health crisis, such as vaccination refusal or hesitancy. Additionally, it allows us to develop a digital communication strategy that can be tailored and used during future crises.

## 2. Literature Review

### 2.1. Comments, Sharing, and Interpersonal Communication

The heuristic-systematic information processing model (HSM) suggests that users engage more with topics they are familiar with and highly involved in [18]. Based on that, we suggest that users engage more with COVID-19-related stories than control-neutral stories due to their familiarity with and high involvement with the pandemic. A post’s content can influence user evaluation of information and communication behavior. False information, conspiracy theories, and mistrust of the government guided online communication during the COVID-19 pandemic [19]. In Israel, there was a greater online engagement with COVID-19 rumors that provided information on how to combat the virus while citing the sources of such information to preserve credibility [20]. Compulsory use of social media correlates with engagement with conspiracy theories and misinformation [14], including the coronavirus infection and vaccination, prejudices against ethnic groups, the virus origin (e.g., 5G towers), and pseudoscientific treatments as a cure [21].

Interpersonal news sharing is crucial during crises due to altruism, providing information and comfort. It can also occur to create an emotional connection. People with strong negative emotions tend to share news to cope with their emotional reactions to news coverage [22,23,24,25,26]. The above review led us to hypothesize the following:

**H1.**  *The identity of the source, which appears in a personal story on a Facebook post about a COVID-19 story (versus control), will affect the willingness to engage with it (H1a) in interpersonal communication (H1b) on social media and (H1c) commenting.*

The source of a message can influence how we respond to it. The heuristic-systematic model (HSM) suggests that people rely on heuristic processing when evaluating content if they are not motivated or unable to assess the quality of the content [18]. Heuristic processing relies on cues, such as the source’s expertise, and requires less cognitive effort than systematic processing to evaluate the information. This aligns with the “principle of least effort,” which states that people use heuristic processing before systematic processing [27]. However, if people are highly motivated and involved, the “sufficiency principle” states that they will use the systematic process [28]. Systematic processing involves integrating cognitive and affective efforts based on evaluating the content of the information [18,29,30,31,32]. In this case, non-expert sources can trigger systematic processing and evaluation of the content instead of the source [30].

The authority heuristic suggests that experts are perceived as more credible and trustworthy than ordinary people when evaluating sources [32,33,34,35,36,37]. The bias hypothesis in the HSM explains that due to heuristic cues, people view messages from authoritative and expert sources as more credible than messages from ordinary people. These cues also affect systematic processing and involve integrating cognitive and affective efforts [18,29,30,31,32]. In contrast, ordinary person sources trigger systematic processing, emphasizing cognitive effort based on the content’s trustworthiness [30]. Different attitudes and behaviors can arise based on how we process information, such as trust or communication behavior. The cognitive trust formed through systematic processing reflects the content of the information [30], whereas the affective trust formed through heuristic processing relates to the heuristic cues, such as the source [30,38].

Consequently, we claim that experts and authoritative sources lead to engagement through cognitive and emotional processing, increasing the likelihood of sharing content. Ordinary people lead to systematic processing and more content-commenting behavior. Sharing requires more mental effort, while commenting is primarily cognitive. Sharing reflects online self-presentation and involves more significant cognitive and affective effort due to greater visibility [39,40]. Online presence reflects offline selves [41]. Sharing posts on social media generates higher engagement and reflects greater responsibility [40] than commenting. Sharing is a higher level of engagement than commenting [42]. Sharing posts spreads information, while commenting facilitates social interaction [43]. Comments allow users to express opinions, gain insight into other perspectives, and collaborate with like-minded individuals [44]. Comments also provide a platform for citizens to challenge mainstream media or the dominant public sphere [45]. Thus, we hypothesized the following:

**H2.**  *Exposure to a COVID-19 (versus control) story will increase the self-reported intention to share the post in interpersonal communication when the source of information is (H2a) an ordinary person, (H2b) a physician, and (H2c) a politician.*

### 2.2. Belief in Conspiracy Theories and Engagement

Conspiracy theories and beliefs in them have grown in volume and spread over the past decade. Based on [17] (p. 24), we defined conspiracy theories as ‘manipulating and misleading people intentionally to achieve political ends’. Individuals who believe in conspiracy theories are more likely to accept misinformation [46]. Conspiracy theories often go viral on social media [8]. At the same time, people who believe in conspiracy theories tend to spend more time on social media and engage with like-minded individuals online. This can lead to the validation of conspiracy beliefs about current political and health issues (such as COVID-19), opposing the government’s emergency regulations (Georgiou et al., 2020) [47], and vaccination [15]. Previous studies found that those spreading misinformation about COVID-19 were more likely to be addicted to social media, suffer from social media addiction, and ignore public health information [7]. Thus, we hypothesized the following:

**H3.**  *The effect of exposure to a COVID-19 story on the self-reported intention to share the post will be moderated by belief in conspiracy theories. COVID-19 stories will influence participants with lower beliefs in conspiracy theories than those with higher belief levels.*

### 2.3. Distrust and Engagement on Social Media

Conspiracy theories also correlate with low levels of trust in governmental and public institutions [48]. Distrust in institutions leads to the popularity of conspiracy theories [49]. COVID-19 vaccine hesitancy is also rooted in distrust of health and political institutions. Transparent communication is crucial to gaining public trust and increasing vaccination rates [50]. Trust is vital for individuals also to trust content. People who trust others are likelier to trust news content [10,11,12]. Social exchange theory suggests trust is essential for building long-term relationships and exchanging views [51,52]. It can help people express themselves freely [53] and reduce risks when sharing information online [54]. Thus, we hypothesize the following:

**H4.**  *The level of institutional trust will moderate the effect of exposure to a COVID-19 story on the self-reported intention to share the post. A COVID-19 story will influence more participants with high levels of institutional trust than those with lower institutional trust.*

According to the literature review and hypotheses, this paper’s main two research questions are as follows:

RQ1: How can different types of stories affect communication behavior by moderating belief in conspiracy theories and institutional trust?

RQ2: How can different information sources affect communication behavior by moderating the belief in conspiracy theories and institutional trust?

## 3. Method

### 3.1. Participants

One thousand three hundred and forty-nine Israeli adults were recruited by the Israeli research firm Midgam Project (see https://www.midgampanel.com/clients/index.asp, accessed on 14 June 2024) (which specializes in online experiments). Following this, participants were randomly assigned to one of four experimental conditions. We removed participants for the following reasons: failing manipulation or attention tests, straight-lining, completing the survey too quickly or slowly, and poor data. In total, 848 individuals aged 18–74, Mage = 42.73, SD = 15.56, of which 50.2% were women, were included in the data analysis.

To ensure no selection bias affected the finding, we conducted a one-way analysis of variance (ANOVA) to test for differences between the demographic factors of age, gender, and education level in response to the four experimental conditions. As expected, we found no significant differences among the demographic variables. See Table 1 for sample characteristics.

Participants who volunteered to participate in the experiment were compensated in five shekels (US $1.40). The Reichman University’s institutional review board approved the study on 4 November 2021 for compliance with standards for the ethical treatment of human participants.

### 3.2. Procedure

This study examines the impact of one of three sources of information: a politician (authority figure), a physician (expert), and an ordinary person (non-expert) who appeared on a Facebook story related to a controversial issue (COVID-19 vaccination) on the willingness to engage with it. The same name was given to these sources (Roni Cohen) to avoid any potential bise. Yet, we have also added to each source a title that indicates their position as a politician, a physician, and a citizen.

Participants were assigned to one of four experimental conditions: a Facebook story featuring a politician, a physician, or an ordinary citizen, with the fourth condition being a neutral story (as control). The experimental manipulation was written in Hebrew by a Hebrew native-language speaker who later translated the story into English (see Appendix A.1 and Appendix A.2 for translating the experimental manipulation).

Following the experimental manipulation, the dependent variables were measured using the same 10 to 12-minute questionnaire across all four experimental conditions (see measures below). The questionnaire, created by the authors, consisted of 33 questions. It included filter questions that asked the participants about the topics of the story they read, where it was posted, and if it mentioned any vaccination recommendations. In addition, it included questions that were validated in previous studies (see also in measures) about communication behavior, belief in conspiracy theories, and institutional trust. Furthermore, participants were asked to provide their vaccination status, news and information consumption habits, and demographic information, including their political orientation, gender identity, age, socioeconomic status, and education level. Finally, participants were debriefed and thanked for their participation.

### 3.3. Data Analysis

The moderation analyses used [55] the PROCESS Macro for SPSS (version 4). We also used SPSS version 28 for the OLS regressions. The unstandardized ordinary least squares (OLS) coefficients and corresponding standard errors calculated are reported.

### 3.4. Measures

- Dependent variable

The willingness to engage was based on three variables (adapted from [56]) that were rated on a 10-point scale ranging from (0) strongly disagree to (10) strongly agree: (1) Interpersonal communication by asking the participants to rate “how likely are you willing to share the text you read in interpersonal communication with friends and family members?” (M = 5.90, SD = 3.51); Sheering asked the participants to rate “how likely are you willing to share the text you read on social media” (M = 3.84, SD = 3.58); Commenting by asking the participants to rate “how likely are you willing to write a comment about the text you read on social media” (M = 3.80, SD = 3.50). We calculated the willingness to engage as the mean score of these three items (α = 0.81, M = 4.51, and SD = 3.01).

- Moderators

Belief in conspiracy theories was measured by asking the participants to state their belief in four prominent conspiracy beliefs regarding the COVID-19 pandemic, rated on a 6-point scale ranging from (1) strongly disagree to (6) strongly agree (adapted from [57]). We calculated the scale as the mean score (α = 0.83, M = 2.13, and SD = 1.11) of the following items: “Governments around the world and the pharma companies are collaborating to vanish information about COVID-19 vaccination” (M = 2.77; SD = 1.16); “there is no COVID-19 pandemic it is a hoax” (M = 1.36; SD = 0.82); “COVID-19 vaccination is a medical experiment” (M = 2.51; SD = 1.16); and “COVID-19 vaccination is more dangerous than the various itself” (M = 1.90; SD = 1.27). (for the distribution of the items, see Table 2).

Institutional trust was measured using four items (adapted from [58]) using a 4-point scale ranging from (1) strongly disagree to (4) strongly agree. The institutional trust scale is based on the mean score of trust in four institutions (α = 0.88, M = 2.20, and SD = 0.80) by asking respondents how much they trust the following: (1) “the government medical experts” (M = 2.63; SD = 0.93); (2) “the minister of health” (M = 2.13; SD = 0.95); (3) “the government finance experts” (M = 2.03; SD = 0.90); and (4) “the prime minister” (M = 2.0; SD = 0.97).

**Control variables**. Political identity was measured on a scale between 0 (right-wing) and 10 (left-wing). Gender was measured on a binary scale, 0 (male) and 1 (female), and age and education were indicated according to the number of years. Religiosity was measured by a 4-point scale ranging from 1—ultra-Orthodox, 2—Religious, 3—Traditional, to 4—secular. The income was measured on a 5-point scale ranging from 1—below 4000 NIS a month to 5—more than 16,001 NIS a month.

## 4. Findings

Table 2 shows that participants had low levels of institutional trust (M = 2.19, SD = 0.80); only 24% indicated high levels of institutional trust (ratings of 3 and 4 on the 1–4 scale). Participants also reported a low level of belief in conspiracy theories (M = 2.13, SD = 1.11), with only 14.4% indicating that they have a high belief in all five conspiracy theories (ratings of 4, 5, and 6 on the 1–6 scale) (Findings supported by the election results in October 2022, where the anti-vaccine party got only small percentage of the general voting population). In addition, we found a high level of willingness to share the personal story in an interpersonal conversation (M = 5.90, SD = 3.51) and lower levels of willingness to share it on social media (M = 3.84, SD = 3.58) or commenting (M = 3.79, SD = 3.50).

### 4.1. The Effect of Exposure to a Personal Story on Engagement

Table 3 presents a one-way ANOVA used to test the main effect of the COVID-19 (versus control) story on the willingness to engage with it in interpersonal communication and on social media. The results indicate there is a significant main effect of the story type(F_(1, 847)_ = 9.23, *p* < 0.05) on the intention to share the story in interpersonal communication; however, no significant main effect was found for intentions to share it on social media (F_(1, 847)_ = 0.29, *p* = n.s.) and to write a comment (F_(1, 847)_ = 0.17, *p* = n.s.) (see Table 3).

To test our first research question and hypothesis (H1) that exposure to a personal health story will increase the intention to share the story (H1a) in interpersonal communication, (H1b) on social media, and (H1c) commenting, we conducted three OLS regression models (see Table 4). Model 1 presented a significant effect of the health story (versus control) on the intention to share the story in an interpersonal conversation (b = 0.10, *p* < 0.001). The regression model also showed a significant association of age (b = 0.13, *p* < 0.001) and education (b = 0.07, *p* < 0.05) with the story type. However, we found no significant effect on the intention to share the story on social media (b = 0.00, *p* = n.s.; model 2) and on writing a comment on the story (b = 0.01, *p* = n.s.; model 3).

Table 5 presents a one-way ANOVA to test the main effect of exposure to one of the three sources (a politician, a physician, or a citizen) featured in a personal health story on Facebook on the intention to share the story. The results indicated a significant main effect of the source of information (F_(3, 847)_ = 3.08, *p* < 0.05) on the intention to share the story in interpersonal communication; however, no significant main effect was found on the intention to share the story on social media (F_(3, 847)_ = 0.16, *p* = n.s.) and to write a comment (F_(3, 847)_ = 1.25, *p* = n.s.).

To test our second research question and hypothesis (H2) that exposure to the personal story of (H2a) a citizen, (H2b) a physician, and (H2c) a politician will increase the intentions to share the story on social media and in interpersonal communication, we conducted an additional three OLS regression models (see Table 6). The results indicated a significant direct effect of the three sources of information, a citizen (b = 0.13, *p* < 0.05), physician (b = 0.17, *p* < 0.05), and politician (b = 0.12, *p* < 0.05), as well as education (b = 1.97, *p* < 0.05) and age (b = 3.53, *p* < 0.001), on the intention to share the story in interpersonal communication. However, no significant effect was found for the intention to share the story of the three sources of information on social media: for a citizen (b = 0.01, *p* = n.s.), a physician (b = −0.01, *p* = n.s.), a politician (b = −0.01, *p* = n.s.; model 2) and for the intention to write a comment on the story of a citizen (b = 0.05, *p* = n.s.), physician (b = 0.03, *p* = n.s.), and politician (b = −0.03, *p* = n.s.; model 3)

### 4.2. Belief in Conspiracy Theories as a Moderator

As is in Ref. [55], Model 1 was used to test our third hypothesis (H3) regarding the moderation effect of belief in conspiracy theories (W) on the effect of exposure to a COVID-19 story (X) regarding the intention to share it (H3a) in interpersonal communication, (H3b) on social media, and (H3c) in comments.

**Figure 1:**
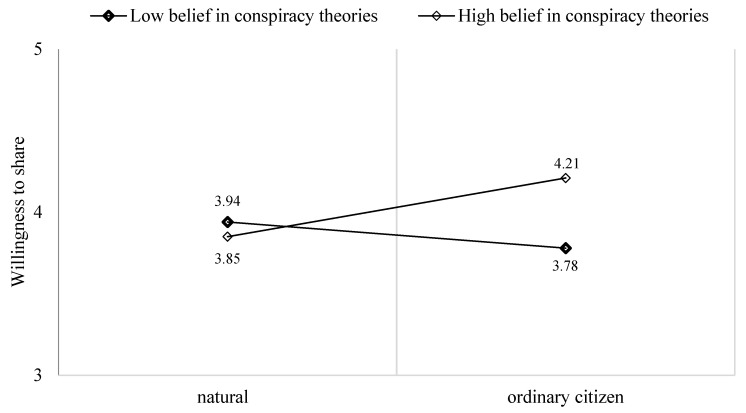
The moderation effect of beliefs in conspiracy theories on the effect of exposure to a citizen’s personal story on the willingness to write a comment.

The results partly supported our hypothesis, demonstrating a significant moderation effect of belief in conspiracy theories on the effect of exposure to a personal story on the intention to share it in interpersonal communication (b = −0.86, SE = 0.33, *p* < 0.05, CI 90%: [−1.51, −0.21]. However, there were no significant results regarding the intention to share on social media and in comments (see Figure 1).

**Figure 2:**
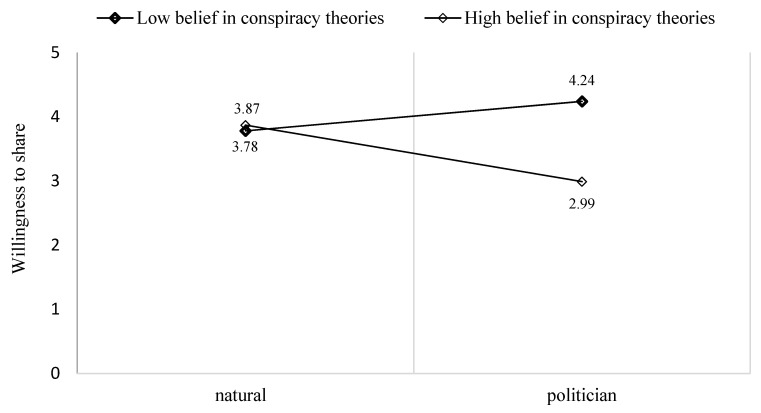
The moderation effect of beliefs in conspiracy theories on the effect of exposure to a politician’s personal story on the willingness to write a comment.

The results demonstrated a significant interactive effect of belief in conspiracy theories and exposure to a personal story on the intention to write a comment on social media when the source is a citizen (b = 0.44, SE = 0.22, CI 90%: [−0.00, 0.88]; Figure 1) and when the source is a politician (b =− 0.59 SE = 0.25, CI 90%: [−1.10, −0.08]; Figure 2). However, a non-significant effect was found when the source was a physician. Participants with higher levels of belief in conspiracy theories tended to engage more with a post published by a citizen compared to those that a physician or a politician had published.

### 4.3. Institutional Trust as a Moderator

As is in Ref. [55], Model 1 was used to test our fourth hypothesis (H4) regarding the moderation effect of institutional trust (W) on the intention to share it (H4a) in interpersonal communication, (H4b) on social media, and (H4c) in comments.

**Figure 3:**
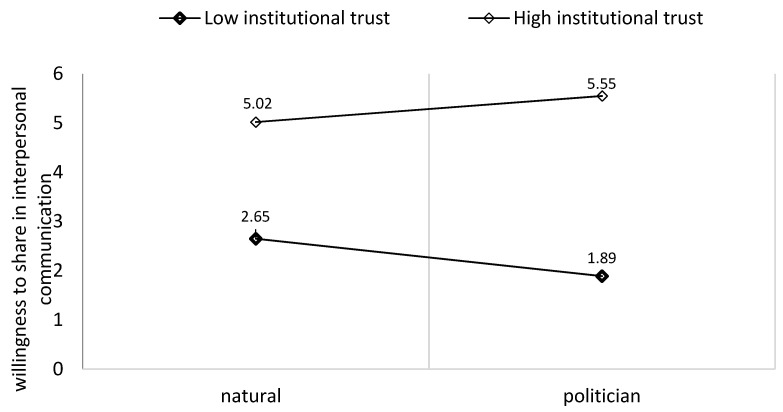
The moderation effect of institutional trust on the effect of exposure to a politician’s personal story on the willingness to share in interpersonal communication.

**Figure 4:**
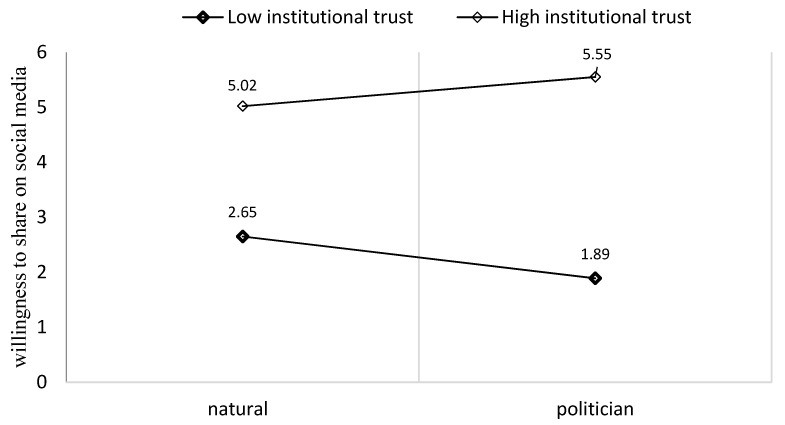
The moderation effect of institutional trust on the effect of exposure to a politician’s personal story on the willingness to share on social media.

The results partly supported our fourth hypothesis, demonstrating a significant moderation effect of institutional trust on the effect of exposure to a personal story on the intention to share in interpersonal communication (b = 1.24, SE = 0.44, CI 90%: [0.38, 2.11]; see Figure 3), or to share on social media (b = 1.73, SE = 0.46, CI 90%: [0.83, 2.64]; see Figure 4). However, commenting on social media did not have a significant effect.

The results partly supported our hypothesis, demonstrating a significant moderation effect of institutional trust on the effect of exposure to a personal story on the intention to share a politician’s personal story in interpersonal communication (b = 0.70, SE = 0.34, CI 90%: [0.02, 1.38]; see Figure 3). Non-significant effects were found when a citizen or a physician posted the story. Participants with higher levels of institutional trust tended to engage more with a post published by a politician compared to those that a physician or a citizen had published.

As is in Ref. [55], Model 1 demonstrated a significant moderation effect of institutional trust on the effect of exposure to a politician’s personal story regarding the intention to share it (b = 0.71, SE = 0.36, *p* < 0.05, CI 90%: [0.00, 1.42]; see Figure 4). However, there were no significant effects for citizen and physician sources. The results did not support the fourth hypothesis on the willingness to write a comment for a personal story of a citizen, a physician, or a politician. Participants with higher levels of institutional trust have a higher level of engagement with politicians’ personal stories on Facebook.

## 5. Discussion

Our findings indicated that users were more likely to share personal health stories in interpersonal communication than control stories. The HSM’s sufficiency principle proposes that users engage more with health crisis-related stories due to their familiarity and high involvement with the pandemic [18,28]. In addition, face-to-face interaction is crucial during times of crisis, especially when dealing with negative emotions like fear and anxiety [22,23,24,25,26].

In addition, we compared the effect of personal stories from authoritative (i.e., a politician) experts (i.e., a physician) and ordinary people (i.e., non-experts). Political stories affect the intention to share a personal story via interpersonal and mediated communication moderated by the level of institutional trust. Following the authority heuristic, authority and expert sources are perceived as more credible [32], as their authority and expertise provide greater trust and reliability [33,34,37]. According to the HSM’s bias hypothesis, people believe messages from experts more than ordinary people because they perceive them as more credible. These cues also affect the systematic processing of the content, which involves integrating cognitive and affective efforts [18,29,30,31,32].

On the other hand, we used ordinary person personal stories, which affected users’ intention to comment on stories moderated by belief in conspiracy theories. An ordinary person might serve as a cue that activates systematic processing, emphasizing cognitive effort based on content trustworthiness [30]. It can result in various attitudes, such as trust or, in this case, communication behavior. The trust formed through systematic processing is characterized as cognitive trust that reflects the content [30], whereas heuristic processing embodies affective trust based on heuristic cues, the message’s source [30,38,59]. Consequently, exposure to politician and physician stories, authority, and expert sources affects sharing behavior, which involves systematic cognitive and heuristic-emotional processes. The intention of users to share content is moderated by trust in officials and institutions. Citizens’ stories, however, lead to systematic cognitive processing moderated by conspiratorial beliefs and a greater intention to comment. Sharing and commenting differ because sharing requires more cognitive and mental effort while commenting is primarily cognitive. Compared to commenting, sharing is considered a higher level of engagement [39,40]. Trust is essential for sharing information [51]. More trustworthy individuals express their opinions more freely [52,54]. People used posts to share vaccine-related information, while comments allowed individuals to debate vaccination issues and challenge prevailing vaccination discourses [44,45]. Believers in conspiracy theories tend to be skeptical of official explanations and are more intent on expressing their opinions online, such as by commenting. However, people with high institutional trust are more likely to share information online since sharing gains greater visibility than commenting [60], indicating their sense of responsibility [40].

The study is not without limitations. Considering Israel’s unique socio-political context, the findings may not be universally applicable. Some marginalized groups within Israeli society (such as ultra-Orthodox) engage less with social media, have lower institutional trust, and believe more in conspiracy theories. Other research methods are required to better understand this phenomenon among marginalized groups. Further research is needed to identify healthcare providers’ role in public health communication strategies and understand how they can contribute more effectively.

In conclusion, a user’s prior trust or belief in conspiracy theories influences their choice of information sources, whether from political or ordinary citizens. These pre-existing beliefs impact the perceived credibility of the source and the level of trust placed in it. Consequently, this study highlights how this affects the user’s likelihood of sharing the story in interpersonal conversations or online platforms. In conclusion, our research findings have the potential to significantly impact the work of strategic communicators, governments, politicians, and health experts, giving the central role of communication in health and vaccination behavior [61]. By tailoring information to users’ attitudes and beliefs, our research can help these professionals better connect with the public, combat conspiracy theories, and improve information dissemination during health crises. Politicians and health experts can also use our research to engage in online discussions and share critical information during emergencies. Effective communication strategies must leverage online and offline channels to connect with the public effectively.

## Figures and Tables

**Table 1:** Sample characteristics (N = 848).

Characteristics	N (%)
Gender	
Men	420 (49.5)
Women	427 (50.4)
Religious affiliation	
Haredi	166 (19.6)
Religious	97 (11.4)
Traditional	142 (16.7)
Secular	443 (52.2)
Political view	
Left	40 (4.7)
Right	95 (11.2)
Center	713 (84.1)
SES	
Less than 400 NIS	61 (7.2)
4001–8000 NIS	112 (13.2)
8001–12,000 NIS	193 (22.8)
12,001–16,000 NIS	187 (22.1)
16,001 NIS or higher	171 (20.2)
Refuse to answer	124 (14.6)
	M (SD)
Age (in years)	42.73 (15.57)
Education (in years)	14.9 (3.9)

**Table 2:** Percentage of people who believe in conspiracy theories.

Item	%
Governments around the world and pharma companies are collaborating to vanish information about COVID-19 vaccination	31.2
COVID-19 vaccination is a medical experiment	28.2
COVID-19 vaccination is more dangerous than the various itself	10.7
There is no COVID-19 pandemic is a hoax	2.9

Note: The percentage in the table includes those who stated that they believe in conspiracy theories.

**Table 3:** One-way ANOVA for the effect of the story type on willingness for engagement.

	Experimental Conditions	Control
	M	M
Sharing in interpersonal communication	6.04	4.99
	F _(847)_ = 9.23, *p* < 0.05	
Sharing on social media	3.84	3.90
	F _(847)_ = 0.29, *p* = n.s.	
Writing a comment on social media	3.80	3.66
	F _(847)_ = 0.17, *p* = n.s	

**Table 4:** OLS regressions model for the effect of story type on willingness for engagement.

	Model 1	Model 2	Model 3
	β	β	β
Personal (vs. neutral) story	0.10 **	−0.00	0.02
Age	0.13 ***	0.18 ***	0.17 ***
Gender	−0.00	−0.05	−0.05
Education	0.07 *	0.00	0.00
SES	0.03	0.00	−0.04
Political identity	−0.05	−0.13 ***	−0.07
Religiosity			
Haredi	0.03	−0.02	0.00
Religious	0.04	0.01	0.03
Traditional	0.00	−0.00	0.02
Constant	3.29 ***	3.98 ***	4.14 ***
Adj. R^2^	0.03	0.04	0.03
F	(9, 830) = 3.52 ***	(9, 830) = 4.75 ***	(9, 830) = 3.54 ***
N	840	840	840

* *p* < 0.05; ** *p* < 0.01, *** *p* < 0.001. Note: Model 1: willingness to share in interpersonal communication; Model 2: willingness to share on social media; Model 3: willingness to write a comment. *The effect of exposure to different types of stories - COVID-19 versus control*.

**Table 5:** One-way ANOVA for the effect of sources of information on willingness for engagement.

	Citizen	Physician	Politician	Control
	M	M	M	M
Willingness to share in interpersonal communication	6.03	6.07	6.01	4.99
	F _(847)_ = 3.08, *p* < 0.05)			
Willingness to share on social media	3.94	3.83	3.72	3.90
	F _(847)_ = 0.16, *p* > 0.05)			
Willingness to write a comment on social media	4.01	3.89	3.42	3.66
	F _(847)_ = 1.25, *p* > 0.05)			

**Table 6:** OLS regressions model for the effect of sources of information on willingness for engagement.

	Model 1 β	Model 2 β	Model 3 β
Source of information			
Citizen	0.13 *	0.00	0.05
Physician	0.17 *	−0.01	0.03
Politician	0.12 *	−0.01	−0.03
Gender	−0.13	−0.05	−0.05
Political identity	−1.18	−0.13 ***	−0.07
SES	0.03	0.00	−0.05
Education	1.97 *	0.07	0.00
Age	3.53 ***	5.04 ***	0.17 ***
Religiosity			
Haredi	0.03	−0.02	0.00
Religious	0.04	−0.01	0.03
Traditional	0.35	−0.00	0.02
Constant	3.29 ***	3.96 ***	4.11 *
Adj. R^2^	0.02	0.04	0.03
F	(11,839) = 2.88 ***	(11,839) = 3.90 ***	(11,839) = 3.18 ***
N	840	840	840

* *p* < 0.05; *** *p* < 0.001. Note: Model 1: willingness to share in interpersonal communication; Model 2: willingness to share on social media; Model 3: willingness to write a comment.

## Data Availability

The original contributions presented in the study are included in the article. Further inquiries can be directed to the corresponding author.

## Appendix A

### Appendix A.1. A Personal Story Posted on Facebook by a Citizen/Physician/Politician (Three Experimental Conditions)

“I have trouble breathing and speaking,” said [Roni Cohen/Dr. Roni Cohen/MK Roni Cohen] today, who was infected with COVID-19 last week and is hospitalized in severe condition at a hospital in the center of the country. According to him, he suffers from several different diseases. Because of their complexity, he was afraid to get vaccinated against COVID-19 since he did not know whether or how the vaccine would affect his body. “I regret not getting vaccinated today because I know the consequences of the Coronavirus are much more serious. Therefore, I urge everyone who has not yet been vaccinated to get vaccinated. If you want to avoid infecting yourself as I did, do not wait until you become infected. Throughout the day, I see the medical teams helping patients. Unfortunately, in some instances, it is already too late. It is not always possible for medical treatment to be successful. The vaccine saves lives”. Meanwhile, to curb the current wave of morbidity, the Ministry of Health has expanded the vaccination campaign and ordered additional vaccines for Israel intended for adults and children. The Ministry of Health urges everyone who is eligible and has not been vaccinated to get vaccinated. It has been proven that the vaccine is the most effective means of combating Coronavirus, and it is accessible to everyone.

### Appendix A.2. A Facebook Story about Apple Trees and Their Consumption (the Control Condition)

During the Tishrei holidays, apple consumption and demand jumped by about 80%, according to a “Measurement and Evaluation” survey company survey. As compared with last year, apple consumption jumped by 90%. In addition to local production, apples are imported through international trade agreements and private imports by traders. In the Galilee and Golan mountains, most orchards are planted since the high mountains’ chill improves the quality and appearance of the fruit. The apple-growing industry is essential in the economies of Galilee and Golan. Several studies have been conducted to improve apple varieties, shape apple trees for picking, and protect and treat fruit after picking. Also, different cultivation methods were brought to Israel worldwide, making picking apples without human assistance possible in the future. Currently, apple tree yields are relatively low, and they are also affected by changes in the weather. Moreover, the fruit only sometimes meets the trade requirements and only pays back the investment cost of the farmer. Since most of the livelihood in the agriculture industry in Galilee and Golan is from the apple orchards, researchers are trying to figure out how to improve the yield and quality of the products and develop the ability to prune and pick apples with robots.
